# Public Health Round-up

**DOI:** 10.2471/BLT.18.011218

**Published:** 2018-12-01

**Ramping up the Ebola response**

A health worker monitors glove sterilization at the Mangina Ebola Treatment Centre, North Kivu in the Democratic Republic of the Congo.

**Figure:**
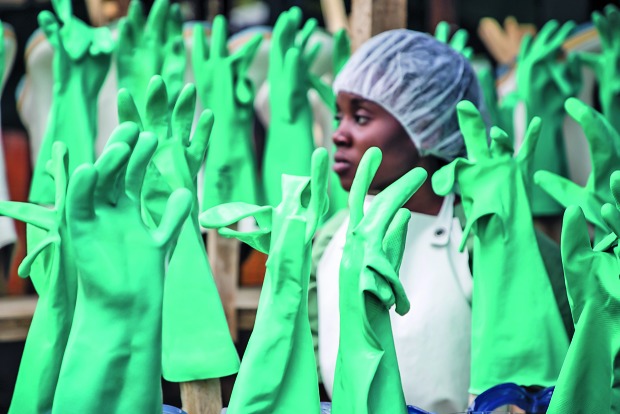
Photographer: Carl Theunis/MSF

## Ebola vaccination in Uganda

Vaccination of front-line health workers in Uganda with the rVSV-Ebola vaccine got under way last month.

Uganda shares a border with the Democratic Republic of the Congo, where an outbreak of Ebola virus disease has claimed 209 lives, for a total 333 reported cases, as of 11 November.

The Ugandan health ministry with support from the World Health Organization (WHO) started vaccination in five districts, Bundibugyo, Kabarole, Kasese, Ntoroko and Bunyanga on the border with the Democratic Republic of the Congo.

A total of 2100 doses of the rVSV-Ebola vaccine were to be administered to the health workers to protect them against Ebola virus, which is circulating in some parts of the Democratic Republic of the Congo and not, so far, been reported in Uganda.

With large numbers of people moving across the Democratic Republic of the Congo–Uganda border, the risk of cross-border transmission of Ebola to Uganda is very high, according to WHO’s risk assessment.

Several studies show that the rVSV-Ebola vaccine is safe and protective against the Ebola virus, but more research is needed before it can be licensed. That is why the vaccine is being administered in the Democratic Republic of the Congo on a compassionate basis to protect people with the greatest exposure to the outbreak, provided recipients give their consent. 

WHO is also supporting neighbouring South Sudan’s health ministry in its training of a 214-member Rapid Response Team at the national level and in neighbouring countries.

The training is part of efforts to strengthen the country’s preparedness capacities and mitigate the risk of importing Ebola virus disease from the North Kivu and Ituri provinces of the Democratic Republic of the Congo.

Other risk mitigation measures include routine screening of all travellers entering South Sudan from the Democratic Republic of the Congo or Uganda by land or air.

A total of 39 points of entry in the high–risk countries have been earmarked to serve as screening sites, 14 of which were fully functional last month. 

WHO is supporting two of the major screening sites: Juba International Airport and the border crossing outside the town of Nimule. Several partners are working to establish isolation facilities in the country.

https://afro.who.int/news/uganda-vaccinates-front-line-health-workers-against-ebola

## UN Security Council Ebola resolution

The United Nations (UN) Security Council called on all armed groups in Ebola outbreak zones in the Democratic Republic of the Congo to respect international law. 

In Resolution 2439, the Security Council seeks to ensure full, safe, immediate and unhindered access for humanitarian and medical personnel, and their equipment, transport and supplies to the affected areas.

The Democratic Republic of the Congo is battling an Ebola outbreak in North Kivu and Ituri, two densely populated provinces. Both provinces have been subject to attacks by different armed groups and are the location of a humanitarian crisis affecting over one million internally displaced people. 

Meanwhile refugees are moving to neighbouring countries, including Uganda, Burundi and the United Republic of Tanzania.

The Security Council condemned the attacks by armed groups in the region and urged the government in the Democratic Republic of the Congo and in neighbouring countries to continue efforts to address and resolve the wider political, security, socioeconomic and humanitarian consequences of the Ebola outbreak.

WHO revised its risk assessment for the outbreak, at national and regional levels, from high to very high on 28 September. 

WHO advises against travel and trade restrictions and assesses the risk of global spread to be low.

https://www.un.org/press/en/2018/sc13559.doc.htm

## Measles and rubella 

Singapore has eliminated measles, and Australia, Brunei Darussalam and Macao Special Administrative Region, China, have eliminated rubella as public health problems, WHO announced on 31 October.

The news came one year after a significant ramping up of investment in immunization programmes, disease surveillance and laboratory capacity in WHO’s Western Pacific Region.

In 2017, cases were already dropping with a reduction in cases for both diseases to the lowest levels on record for the Region: 5.2 cases of measles and 2.45 cases of rubella per million people.

Both viruses are highly contagious, but their spread is preventable with safe and cost-effective vaccines. 

Measles can cause pneumonia, blindness and brain damage and death in children. Rubella is particularly serious for pregnant women, with infections sometimes leading to miscarriage or birth defects, including blindness, deafness and heart disease. 

Historically low levels of measles were recorded in the WHO Western Pacific region in 2012, then a resurgence of cases and deaths occurred from 2013 to 2016. There are now nine measles-free countries and areas in the WHO region, five of which have also stopped transmission of rubella.

http://www.who.int/laos/news/detail-wpro/31-10-2018-singapore-wipes-out-measles-australia-brunei-darussalam-and-macao-sar-(china)-eliminate-rubella

**Cover photo**

A mother with newborn twins at the kangaroo mother care ward at the government hospital in Nalgonda District in Telangana State, India.

**Figure:**
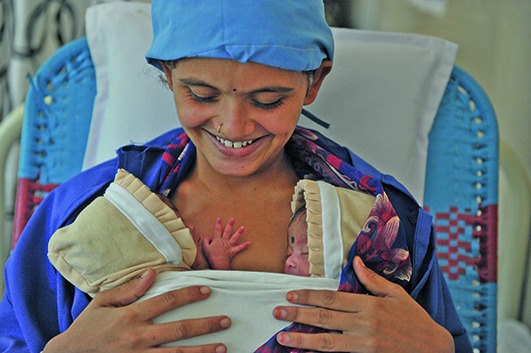
UNICEF/UN0135389/Selaam

## SAGE warning on immunization trends

The Strategic Advisory Group of Experts (SAGE) on Immunization has warned that many of the targets set out in the Global Vaccine Action Plan 2011–2020 are unlikely to be attained by the end of the decade. 

Meeting in Geneva on 23–25 October, the group drew attention to recent outbreaks as a reminder that countries should continue to invest in immunization, and provided guidance on the use of unlicensed vaccines in emergencies, including vaccines for Ebola virus. 

http://www.who.int/immunization/policy/sage/summary_sageoct18_report.pdf?ua=1

## Physical health and mental disorders

WHO launched new guidelines last month that recommend integrated care for people living with severe mental disorders. 

People living with schizophrenia and other psychotic disorders, bipolar disorder, and moderate-to-severe depression have a 2–3 times higher average premature mortality rate compared to the general population, translating to a reduced life expectancy of 10 to 20 years. 

While deaths due to accidents, homicide, or suicide occur with greater frequency for this group than for the general population, most premature deaths are attributable to noncommunicable and communicable diseases.

The guidelines seek to address this problem by focusing on the need to treat the whole person and not only the disorders. 

The guidelines aim to facilitate the implementation of WHO’s Comprehensive Mental Health Action Plan and the Mental Health GAP Action Programme.

http://www.who.int/mental_health/evidence/guidelines_severe_mental_disorders_web_note_2018/en/

## Malnutrition double burden 

Policy-makers, researchers, health professionals and representatives from civil society gather this month to discuss the double burden of malnutrition at the International Atomic Energy Agency (IAEA) headquarters in Vienna, Austria.

Malnutrition is characterized by undernutrition (stunting, wasting, vitamin and mineral deficiency) and overweight, obesity or diet-related noncommunicable diseases. 

The symposium presented evidence regarding the global magnitude of the problem, evidence on biological pathways through which early nutrition influences noncommunicable diseases, and the role of stable isotope techniques and new tools in assessing the double burden. 

Knowledge gaps and research needs were identified, as were relevant actions and policies.

The symposium was organized by the IAEA, WHO and the United Nations Children's Fund on 10–13 December 2018 to strengthen understanding of the double burden. 

https://www.iaea.org/events/understanding-the-double-burden-of-malnutrition-symposium-2018

## Call for virginity testing ban

The United Nations Human Rights Council, the United Nations Entity of Gender Equality and the Empowerment of Women and WHO made a global appeal in October to end virginity testing on women. 

Virginity testing is often performed by inspecting the hymen for tears or its size of opening, and/or inserting fingers into the vagina. 

Both techniques are practiced under the belief that the appearance of the female genitalia can indicate a girl’s or woman’s history of sexual activity. WHO states that there is no evidence that either method can prove whether a woman or girl has had vaginal intercourse or not.

Virginity testing has been documented in at least 20 countries worldwide. Women and girls are often forced to undergo virginity testing at the request of parents or potential partners to establish marriage eligibility or at the request of employers for employment eligibility. 

The virginity tests are performed mainly by doctors, police officers or community leaders. 

http://www.who.int/news-room/detail/17-10-2018-united-nations-agencies-call-for-ban-on-virginity-testing

**Looking ahead**

28 January – 5 February - 144th session of the Executive Board WHO headquarters.

29 January – 3 February - The Prince Mahidol Award Conference, Bangkok, Thailand. Theme: The Political Economy of NCDs: A Whole of Society Approach.

11 February – Meeting on global mental health advocacy and communications WHO headquarters.

12 –13 February – The First Food and Agricultural Organization/WHO/African Union International Conference on Food Safety.

